# Extrahepatic Autoimmune Diseases are Prevalent in Autoimmune Hepatitis Patients and Their First-Degree Relatives: Survey Study

**DOI:** 10.2196/ijmr.9625

**Published:** 2018-12-19

**Authors:** Rachel Fogel, Megan Comerford, Prianka Chilukuri, Eric Orman, Naga Chalasani, Craig Lammert

**Affiliations:** 1 Division of Gastroenterology and Hepatology Indiana University Indianapolis, IN United States

**Keywords:** autoimmune hepatitis, first-degree relatives, social media

## Abstract

**Background:**

Concurrent autoimmune illnesses contribute to increased medical burden and reduced quality of life in patients with autoimmune hepatitis (AIH). The frequency of coexisting autoimmune conditions among North American patients with AIH and their families remains incomplete. Challenges associated with disease capture in the electronic medical record, high study costs, and geographic spread of patients are formidable barriers to understanding the extent of concurrent autoimmune conditions in these groups.

**Objective:**

This objective of this study was to examine the frequency of extrahepatic autoimmune diseases (EHAD) among AIH cases and healthy controls as well as their first-degree relatives using social networking sites (SNS).

**Methods:**

We developed a 53-question survey detailing the history of autoimmune diseases. A survey link was posted at routine intervals within specific Web-based cohorts on SNS. Healthy controls, without self-reported autoimmune liver disease, were recruited from Amazon’s Mechanical Turk. Continuous variables were summarized using medians and P values obtained with the Wilcoxon rank-sum test. Categorical variables were compared using the chi-square test.

**Results:**

Compared with controls (n=1162), cases (n=306) were more likely to be older (median age: 49 vs 33 years), female (284/306, 92.81% vs 955/1162, 82.18%), and have an EHAD (128/306, 41.83% vs 218/1162, 18.76%; *P*=.001). The most frequent EHADs among cases were thyroid disease (49/306, 16.01% ), Sjögren syndrome (27/306, 8.82%), Raynaud phenomenon (23/306, 7.52%), and psoriasis (22/306, 7.19%). Overall, 55.88% (171/306) of cases and 35.71% (1601/4484) of controls reported at least 1 first-degree relative (FDR) with a history of EHAD (*P*=.001). Cases had a significantly higher risk of EHAD than controls after the adjustment for age, sex, race, and body mass index: odds ratio 2.46 (95% CI 1.8-3.3); *P*=.001.

**Conclusions:**

Patients with AIH report higher prevalence of coexistent EHAD than healthy controls, and their FDRs are also more likely to have autoimmune disorders.

## Introduction

Autoimmune hepatitis (AIH) is characterized by T-cell-mediated inflammation of the liver and typical autoantibodies [1,2]. If left untreated, AIH can result in progressive liver disease, including cirrhosis and failure, often requiring liver transplantation [3]. Several epidemiological studies have yielded a wide array of incidence globally, with rates of 0.08 per 100,000 in Japan [4] to 43 per 100,000 among Alaska natives [5]. Regardless of the geographical location, AIH is known to affect women primarily and the age of onset appears to be bimodal [6].

The etiology of AIH remains unclear, but both environmental and genetic factors have been hypothesized despite few supporting studies. Environmental contributions are supported by varying global AIH incidence rates [4-6], evidence of both drug-induced and viral-induced AIH [7-9], and disease risk associated with certain exposures [10]. Genetic risk associations have been observed predominantly at the human leukocyte antigen (*HLA*) locus [11] and in at least one non-*HLA* gene [12]. Beyond limited genome-wide data and prior candidate gene studies, descriptive European reports support an underlying autoimmune phenotype, as observation of extrahepatic autoimmune diseases (EHAD) occurring concurrently with AIH has been as high as 42% [6,13,14]. To date, only 2 European studies have examined the family history of EHAD in patients with AIH by collecting survey data and retrospectively reviewing patient records [6,14]. Furthermore, a case-control assessment of EHAD among patients with AIH and their first-degree relatives (FDR) remains incomplete.

Geographic barriers and lack of a nationwide medical record system have limited AIH investigation; however, the advent of Web-based social networking sites (SNS), such as Facebook and Twitter, have bridged this gap in research accessibility [15]. Web-based patient groups can now provide an integrative, synergistic system for collecting data and engaging patients in the participation and advancement of research [16,17]. This study aims to utilize a novel research method centered on SNS to examine the association between AIH and the presence of autoimmune diseases among patients with AIH and their FDRs.

## Methods

### Social Networking Sites

We implemented an AIH patient recruitment method using SNS for this study. We have previously described the advantages of these platforms to provide patients with AIH with access to health information, patient-directed support, and opportunities for research involvement [18]. The Autoimmune Hepatitis Research Network, a private Facebook group created and managed by a physician-led research team at Indiana University, was created in 2014 and currently hosts nearly 1800 members. Patients with AIH (cases) were recruited to participate through monthly electronic study advertisements over a 6-month study period (June 2015-January 2016) on the Autoimmune Hepatitis Research Network, as well as the Autoimmune Hepatitis Association public Facebook page. In addition, the study team posted a research invitation on associated Twitter accounts monthly (@craiglammertIU: 218 and and @AIH_Association: 367 followers, as of June 2017)] during the study period. Advertisements included disease background information, inclusion and exclusion information, and a direct survey link. For participation, cases were required to be aged ≥18 years and have previously received a diagnosis of AIH from a medical doctor.

### Acquisition of the Control Population

Controls without self-reported AIH were screened and recruited from Amazon’s Mechanical Turk (MTurk), a crowdsourcing website for the completion of requester-directed tasks, which has been shown to approximate the demographics of American adults over the Web [19]. MTurk workers with high approval ratings have been shown to answer attention check questions correctly, thereby minimizing the selection bias [20]. Thus, to obtain high-quality data, participation was limited to high- reputation MTurk workers with a 95% approval rating or above. A survey link was posted to MTurk daily, and MTurk workers completed the survey in exchange for a small monetary reward. Controls were required to be aged ≥18 years, US residents, and report no history of autoimmune liver disease. The controls received US $0.25 compensation for their participation and were only able to complete the survey once. The Indiana University Institutional Review Board approved all study methods.

### Questionnaire Development

**Figure 1:**
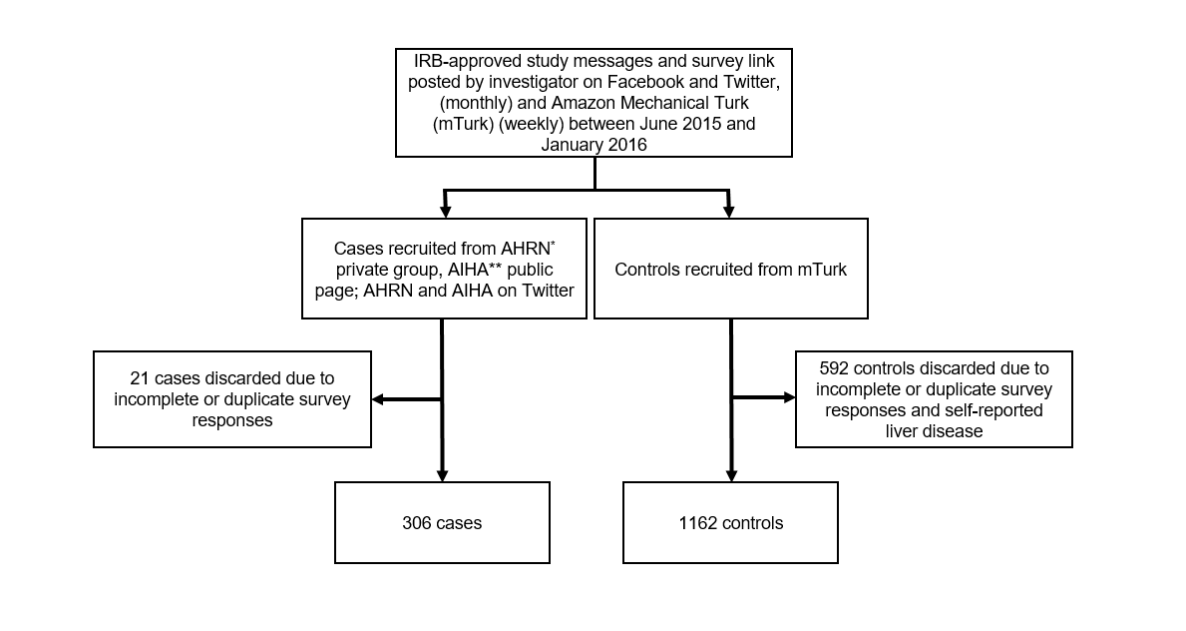
Electronic recruitment, exclusion, and inclusion of study participants. IRB: international review board, AHRN: Autoimmune Hepatitis Research Network, AIHA: Autoimmune Hepatitis Association.

An autoimmune disease questionnaire was created using Web-based survey development software and included key demographics, as well as 48 questions addressing personal and FDR medical history of other autoimmune diseases. The questionnaire was adapted from the Mayo Clinic Medical Questionnaire for Chronic Cholestatic Liver Diseases and took approximately 10 minutes to complete [21]. We assessed 12 common EHAD and 3 hepatic autoimmune diseases, including AIH, primary sclerosing cholangitis (PSC) and primary biliary cholangitis (PBC). A total of 2081 responses to the survey were collected over the 6-month study period. Incomplete surveys, duplicate entries, and self-reported liver disease in controls were removed. A total of 1468 participants were included in the study analysis, including 306 cases and 1162 controls (Figure 1).

### Statistical Analysis

Continuous variables were summarized using medians and the 25th and 75th percentiles, and *P* values were obtained with the Wilcoxon rank-sum test. Categorical variables were compared using the chi-square test. Logistic regression was used to estimate the risk of concurrent EHAD and EHAD within FDR after adjusting for age, sex, race, and body mass index (BMI). Statistical analysis was completed using IBM SPSS Statistics software (version 2.0).

## Results

Table 1 presents the demographic characteristics of cases and controls.

Compared with controls, cases were more likely to be female (284/306, 92.81% vs 955/1162, 82.18%), older (49 years vs 33 years, median), Caucasian (266/306, 86.93% vs 944/1162, 81.23%), and have a higher BMI (28.5 vs 25.8). At least 1 EHAD was reported in 41.83% (128/306) of cases and 18.76% (218/1162) of controls (*P*<.001; Table 2).

Autoimmune thyroid disease was the most common EHAD in cases (49/306, 16.01%) and controls (67, 1162, 5.77%). The next most prevalent diseases were Sjögren syndrome (27/306, 8.82%), Raynaud phenomenon (23/306, 7.52%), and psoriasis (22/306, 7.19%) among cases and psoriasis (60/1152, 5.16%), rheumatoid arthritis (46/1162, 4.00%), and type 1 diabetes mellitus (22/1162, 1.89%) among controls. All EHADs were markedly more frequent in cases than controls, except for Crohn disease and type 1 diabetes mellitus (Table 2). Both PBC and PSC were more frequently observed in cases (50/306, 16.34% and 46/1162, 4.00%, respectively) than in controls (2/306, 0.69% and 10/1162, 0.86%, respectively; *P*<.001). Among cases with the AIH-PBC overlap, the most frequent EHAD was autoimmune thyroid disease (12/50, 24%), Sjögren syndrome (8/50, 16%), and psoriasis (7/50, 14%). Cases with the AIH-PBC overlap were more likely than cases without the overlap to have Sjögren syndrome (8/50, 16% vs 15/256, 5.7%; *P*=.05) and lupus (7/50, 14% vs 15/256, 5.9%; *P*=.02). Among cases with the AIH-PSC overlap, the most frequent EHAD was Crohn diseases (3/12, 25%), ulcerative colitis (2/12, 17%), and psoriasis (2/12, 17%). Cases with the AIH-PSC overlap were more likely to have Crohn disease (3/12, 25% vs 3/294, 1%; *P*=.001) and ulcerative colitis (2/12, 17% vs 12/294, 4.1%; *P*=.06) than cases without the overlap. A comparison of cases with and without EHAD revealed similar demographics. Female sex (93.81% vs 92.12% female), age at AIH diagnosis (47 years vs 42 years), race (88.32% vs 86.02% Caucasian) and BMI (29.2 vs 28.3) were similar between cases with EHAD and cases without EHAD, respectively.

Overall, 6080 FDR autoimmune disease histories were obtained, including 1596 FDRs in cases and 4484 in controls. Cases more frequently had an FDR with at least one autoimmune disease compared with controls (892/1596, 55.89% vs 1601/4484, 35.71%; *P*=.001; Table 2). Autoimmune thyroid disease was the most common reported EHAD in case FDRs (318/1596, 19.93%), whereas rheumatoid arthritis was the most common in controls (244/1596, 15.23%). FDR EHADs, such as autoimmune thyroid disease, celiac disease, ulcerative colitis, psoriasis, Raynaud phenomenon, Sjögren syndrome, and type 1 diabetes mellitus, were more prevalent in cases compared with controls (*P*=.001; Table 2). Autoimmune liver diseases were generally infrequent in FDRs of both groups. PSC was the only autoimmune liver disease seen more in FDRs of cases compared with controls (5/1596, 0.31% vs 0/4484, 0%; *P*=.001).

**Table 1:** Demographic characteristics of cases and healthy controls.

Characteristics		Cases (n=306)	Controls (n=1162)	*P* value
Female, n (%)		284 (92.81)	955 (82.18)	<.001
**Age (years)**				
	At the time of study	49.0	33.0	<.001
	At the time of diagnosis	44.0	N/A^a^	N/A
Caucasian, n (%)		266 (86.93)	944 (81.23)	.02
Weight (kg), median		76.0	72.6	.01
Body mass index, median		28.5	25.8	<.001
First-degree relatives, n (total=average #/participant)		1596 (5.2)	4484 (3.8)	N/A
Siblings, n		560	1417	N/A
Children, n		519	874	N/A

^a^N/A: not applicable.

**Table 2:** Concurrent autoimmune diseases among cases and controls as well as first-degree relatives.

Concurrent autoimmune diseases	Cases with condition (n=306), n (%)	Controls with condition (n=1162), n (%)	*P* value	Cases with an FDR^a^ condition (n=306), n (%)	Controls with an FDR condition (n=1162), n (%)	*P* value
Any extrahepatic autoimmune disease	128 (41.83)	218 (18.76)	<.001	171 (55.88)	415 (35.71)	.001
Autoimmune hepatitis	306 (100.00)	0 (0.00)	NS^b^	14 (4.58)	26 (2.24)	NS
Autoimmune thyroid disease	49 (16.01)	67 (5.77)	<.001	61 (19.93)	89 (7.66)	.001
Celiac disease	16 (5.23)	15 (1.29)	<.001	19 (6.21)	33 (2.84)	.007
Crohn disease	7 (2.28)	13 (1.11)	NS	9 (2.94)	41 (3.53)	NS
Ulcerative colitis	12 (3.92)	12 (1.00)	<.001	25 (8.17)	46 (4.00)	.004
Lupus	14 (4.58)	14 (1.20)	<.001	15 (4.90)	33 (2.84)	NS
Multiple sclerosis	1 (0.33)	7 (0.60)	.008	8 (2.61)	33 (2.84)	NS
Primary sclerosing cholangitis	12 (3.92)	100 (0.86)	<.001	1 (0.31)	0 (0.00)	.001
Primary biliary cholangitis	50 (16.34)	80 (0.69)	<.001	5 (1.63)	13 (1.12)	NS
Psoriasis	22 (7.19)	60 (5.16)	.02	48 (15.69)	109 (9.38)	.002
Raynaud phenomenon	23 (7.52)	21 (1.81)	<.001	58 (18.95)	119 (10.24)	.001
Rheumatoid arthritis	19 (6.21)	46 (4.00)	<.001	56 (18.30)	177 (15.23)	NS
Scleroderma	3 (1.00)	30 (0.26)	.003	2 (0.65)	10 (0.86)	NS
Sjögren syndrome	27 (8.82)	50 (0.43)	<.001	30 (4.25)	53 (0.77)	.001
Type 1 diabetes mellitus	3 (1.00)	22 (1.89)	NS	30 (9.80)	53 (4.56)	.001

^a^FDR: first-degree relative.

^b^NS: not significant.

Table 3 shows further assessment of EHAD among specific FDRs. Compared with controls, there was a higher frequency of EHAD among mothers (95/295, 32.2% vs 274/1110, 24.7%; *P*=.01), siblings (82/600, 13.67% vs 120/1799, 6.67%; *P*=.001), and children (36/411, 8.76% vs 40/874, 4.58%; *P*=.004) of cases. In total, only 3.71% (14/306) cases and 2.14% (27/1162) controls reported an FDR with AIH (*P*=.05). Further depiction of AIH per FDR did not reveal any differences between cases and controls, yet AIH within mothers appeared more likely among cases (7/295, 2.37% vs 10/1110, 0.90%; *P*=.06).

A logistic regression model was used to assess the risk of concurrent EHAD and EHAD within FDRs of all participants after adjusting for current age, sex, race, and BMI. After the adjustment, both the odds of EHAD (OR 2.46, 95% CI 1.8-3.3; *P*=.001) and EHAD among FDR (OR 2, 95% CI 1.5-2.7; *P*=.001) were significantly higher among cases than among controls.

**Table 3:** Proportion of autoimmune diseases among first-degree relatives of autoimmune hepatitis cases and healthy controls.

Prevalence of autoimmune disease		Cases	Controls	*P* value
**Extrahepatic autoimmune disease, n (%)**				
	Mother^a^	95 (32.2)	274 (24.7)	.01
	Father^b^	45 (15.3)	130 (11.7)	NS^c^
	Siblings^d^	82 (13.67)	120 (6.67)	.001
	Children^e^	36 (8.76)	40 (4.58)	.004
**Autoimmune hepatitis, n (%)**				
	Mother	7 (2.37)	10 (0.90)	.06
	Father	1 (0.34)	7 (0.63)	NS
	Siblings	6 (1.00)	9 (0.50)	NS
	Children	0 (0.00)	1 (0.11)	NS

^a^Cases: n=295; controls: n=1110.

^b^Cases: n=295; controls: n=1110.

^c^NS: not significant.

^d^Cases: n=600; controls: n=1799.

^e^Cases: n=411; controls: n=874.

## Discussion

This first-ever SNS-supported assessment of EHAD within AIH cases and their FDRs revealed that EHAD is prevalent among both cases and their corresponding FDRs. Specifically, in this study, 41.83% (128/306) of cases and only 18.76% (218/1162) of controls reported an EHAD at the time of the survey completion. Furthermore, 55.88% (171/306) of cases and 35.71% (1601/4484) of controls reported at least one FDR with an EHAD. The assessment of AIH cases with and without EHAD did not demonstrate any demographic differences. The observation of increased EHAD in cases and their FDRs compared with controls remained statistically significant even after adjusting for current age, sex, race, and BMI.

The findings of our case-control study provide a similar estimate of concurrent EHAD in AIH compared with prior descriptive European reports that document the prevalence between 26% and 42% [8,13,14]. In our multivariate model, the odds of an EHAD in cases were almost 2.5 times than that of controls. A detailed comparison of individual autoimmune diseases within cases revealed similar increased EHAD frequencies as previous studies. For instance, we observed autoimmune thyroid disease reported in 16.01% (49/306) of cases, similar to that reported from a large study (9%) of concomitant autoimmune diseases in patients with AIH from the Netherlands [6]. Similarly, both this study and van Gerven et al’s [6] study revealed inflammatory bowel disease, rheumatoid arthritis, and celiac disease occurring within 4%-10% of cases. In addition, we provide evidence that patients with overlap disorders (AIH-PBC and AIH-PSC) maintain a high prevalence of concurrent EHAD typically observed in PBC (Sjögren syndrome and psoriasis) and PSC (inflammatory bowel disease). It should be noted that our study was completed at a low cost and within 6 months by a single study team, while the Dutch study required 31 centers to complete. These findings are a testament to the application of social media tools in medical research, further echoing the sentiment that large studies in rare disease can be completed efficiently and at very low cost.

The observation of increased EHAD within cases is not unexpected, particularly as reports within other autoimmune diseases have described an “autoimmune phenotype,” in which autoimmune diseases frequently coexist in an individual. For example, studies in disorders, such as thyroid disease [22], celiac disease [23], and rheumatoid arthritis [24], have revealed high rates of other concurrent autoimmune diseases. This seemingly frequent observation of disease coexistence may be thematically rooted in the *HLA* region on chromosome 6. Variable alleles at this locus have been strongly associated with autoimmune disease risk, yet in some instances, other variants provide protection. The first and only AIH-specific genome-wide association study, to date, has confirmed the *HLA* locus at 6p21 as a region of heightened disease risk [12]. Assessments of other single-nucleotide polymorphisms associated with autoimmune disorders were interrogated in the AIH genome-wide association study, revealing trends of association with AIH even after adjusting for single-nucleotide polymorphisms from the HLA. The shared association of risk alleles within a spectrum of organ-specific autoimmune diseases exemplifies inherent pleiotropic effects of associated genes [25]. Future genome-wide studies will likely provide only a few disease-specific associations among autoimmune diseases, and even more; it remains unlikely that these specific risk loci will clarify the organ specificity. Careful investigation of the exposome, rare genetic variants, and epigenome within cases and their FDRs will provide a higher resolution of the disease risk.

Our interrogation of FDRs of patients with AIH remains the largest interrogation ever with >6000 individual histories, as well as the only case-control assessment. We found a high percentage of EHAD within FDR cases (171/306, 55.88%), compared with only 2 other published reports that examined EHAD among FDR (42% in the Netherlands [6] and 16% in the United Kingdom [14]). In fact, the odds of having an FDR with EHAD in our cases was double than that of controls in an adjusted model. We argue that the observed frequency of EHAD in FDR cases in this study is accurate, as patients within the sampled social media groups commonly include more autoimmune disease awareness and familiarity with associated conditions [18]. We also believe case attestation to the diagnosis of AIH is reliable, as we have reported a high degree of concordance between reports of patients with AIH and their reviewed medical records within the same digital communities [26]. Furthermore, the often observed female sex and higher educational attainment in patients with AIH has been associated with the increased uniformity between patient reports and medical records [27]. The low rate of EHAD observed in FDRs in the United Kingdom study is likely artificially low because of the study methodology that was founded in the medical record review [14].

Despite a significant difference of any EHAD between case (171/306, 55.88%) and control (1601/4484, 35.71%) FDRs, close to 50% of the 12 assessed EHADs were no different between the FDR groups. Interestingly, the frequency of 2 autoimmune liver diseases, AIH and PBC, were also similar among case and control FDRs in this study. Further examination of autoimmune liver disease in FDRs within each respective disease may help elucidate distinct pathogenetic disparities or similarities among each (PBC, PSC, and AIH). For instance, Mantaka et al [28] and Jones et al [29] observed that between 6% and 10% of PBC cases have an FDR with PBC, respectively. The lower observed frequency of AIH within case FDRs in this study (3.71%) may represent important disease-specific differences in the genetic penetrance or susceptibility associated with the exposome. Moreover, Jones et al [29] found the highest prevalence of FDR PBC among cases’ daughters (2.3%). In this study, AIH was similarly prevalent in cases’ mothers (2.37%) and siblings (1%), yet these frequencies were not markedly different from controls.

SNS are attractive research tools, and if implemented cautiously, may help reduce large gaps in rare disease research. There are >1.3 billion users on Facebook alone, and many patients use this as an application for medical information and health-related support from peers [30,31]. These applications are perfectly suited to transcend well-described issues in traditional research methods such as high study costs, coordination of multiple centers, and wide geographic patient distribution [18]. For example, this study cost US $667, was performed at a single center, and included patients from all over North America. SNS may not be suitable for all disease populations; however, in AIH, social media is an easy methodological choice, given a significant amount of demographic overlap.

In summary, this study utilized SNS as a low-cost, effective research method to examine the associations between AIH and other autoimmune diseases among patients and their FDR using a case-control study design. Our data maintain the previously observed autoimmune phenotype of patients with AIH and their family members, such that cases and case FDRs were almost twice as likely as controls and control FDRs to have been diagnosed with, at least, one EHAD. These findings lend further support to an inheritable genetic predisposition underscoring the etiology of AIH. Overall, the FDR prevalence of AIH was found to be quite low among cases, and, thus, broad FDR screening for AIH is not indicated. However, female FDRs of patients with AIH, mother-daughter pairs in particular, may be worth educating of associated symptoms, given a slightly higher risk of the disease development.

## Abbreviations

AIH: autoimmune hepatitis
BMI: body mass index
EHAD: extrahepatic autoimmune diseases
FDR: first-degree relatives
*HLA*: human leukocyte antigen
MTurk: Mechanical Turk
PBC: primary biliary cholangitis
PSC: primary sclerosing cholangitis
SNS: social networking sites
